# Government and the Investigation of Contagious Diseases among Domestic Animals

**Published:** 1881-04

**Authors:** 


### EDITORIAL DEPARTMENT.

### GOVERNMENT AND THE INVESTIGATION OF CONTAGIOUS DISEASES AMONG DOMESTIC ANIMALS.

AT this moment the whole country has been awakened by alarming reports, to the effect that disease is rife among some of our most important food-producing animals, and the immediate results appear to be that American pork has been interdicted in France, Germany, Russia, Spain, Italy, and Greece, while other countries are on the point of adopting similar measures. When one thinks that the export values of hogs and their products amounts to over $100,000,000 annually, the importance of the matter to the country at large becomes apparent.

Let us see what steps the general Government has been taking in this and similar directions to give confidence that she exercises a proper supervision over the healthfulness of her exports, or indeed, that she has any useful information that is at the service of her citizens. We turn to the Annual Report of the Commissioner of Agriculture, which bears the imprint of 1880, and presumably gives us the result of all the latest inquiries garnered by the Government officials in their various capacities.

Evidently, hog cholera, our *bête noire* at present, has attracted the most attention, for we find that it consumes ninety pages out of the 268 of the little volume. The *piece de resistance* on this topic is clearly the report of Dr. H. J. Detmers, though, in the words of the introductory preface, it " contains few additional symptoms worthy of mention." Nor did thirty additional *post-mortem* examinations " reveal any new morbid changes worthy of mention." Dr. Detmers, whose work in other directions has entitled him to more than the usual attention accorded a veterinarian of the present epoch in this country, gives a brief review of such facts as are generally known, and then details the results of some special experiments. It is quite evident that he is glad to deal with the microscopic origin of the disease, and the frequency with which he finds the *bacillus suis* in his morbid materials, shows that he has imbibed the zeal of a Hallier, Pasteur, or Salisbury. Unfortunately his drawings do not convey to one's mind the impression that he has dealt with anything but rather wrinkled or bloated specimens of the ordinary bacteria, which he might, perhaps, have found in other places than in the lungs or intestines of hogs, had he thought of looking for them.

Dr. James Law follows in a report which is characterized by the discriminating judgment which makes this observer well fitted to assume the fascinating but dangerous role of an investigator. His experiments in the conveyance of the disease to the sheep and rat, and then the final re-inoculation of the morbid product upon the healthy pig, show a truly scientific acumen, and we may expect from his work some results of definite value. In this direction, certainly, the Government should push investigations with the utmost rigor; for if a modified virus can be obtained that will produce the disease in a less intense form, which will not affect the general health of the animal, and yet grant him immunity from a second attack, we shall have made a discovery that will rank with that of vaccination.

Returning from the reading of this short but suggestive article to take up the next in order, we fairly open our eyes with astonishment, and wonder what next will creep into a government report.

This author, too, is a microscopist, and he plunges at once into the bottom of his subject.

Let us quote him: "With from one to three hundred diameters I find a dark and nucleated cell or cells, with dark outlines and a dark centre. The cell appears circular at first, but in its different stages of aggregation or fermentation it assumes an egg-shape from the piling of one upon another; or a better comparison, perhaps, would be that of a cauliflower or head of curled lettuce.

The tissues of these cells appear ecchymosed." This author has evidently framed his mind to have a theory which shall at least have the merit of originality, and, he adds, naively enough, that his "observations so far overturn the received opinions of this disease."

It is wearisome to criticize a book of which the best has already been written. However, since it must be done, a report follows by Dr. D. E. Salomon, which adds nothing to the subject of splenic fever (Southern cattle fever), but is at great pains to reaffirm or restate certain pathological theories which date back more than a dozen years, and never had any acceptance except from those visionary philosophers that are ready to accept any theory the moment it sees the light.

It is needless to say to those who are informed of these and kindred matters, that the *coniothecium stilesianum* is not currently supposed to cause splenic fever, nor did Billings and Curtis find it when employed by the government to look into the matter.

In the remainder of the volume we find a review of Prof. Leur's book on pleuro-pneumonia, an article on rinderpest, which, by the way, has never yet appeared in this country, so that it can hardly be regarded as of prime importance just now to the farmer or stock breeder; an article on glanders and farcy, that is based on work done nearly twenty years ago, and is taken nearly bodily from a published work; annotations on French experiments; a mass of communications from a few intelligent and a great many illiterate individuals scattered throughout the country, and short items of intelligence, mainly on the diseases of hogs and cattle, from the various States of the Union.

We close the book with positive mortification that a department of the greatest government in the world should publish an official report which contains almost no facts of positive value, though they are manifestly within the reach of intelligent persons; and this in the face of the positive necessity that we should have the information, or else lose the greater part of a trade that has assumed the most colossal proportions, and furnishes support to hundreds of thousands of our citizens.

Doctoring Through the Newspapers. — It is the custom with a number of agricultural papers to have a veterinary department, which is devoted to prescribing for the various diseases of the domestic animals. A farmer's horse or cow, or sheep, or pig is taken sick; he sends on a record of the symptoms to his paper, and it is published in the proper department, with medical advice.

We wish to call the attention of members of the veterinary profession to this practice, and to beg that they use their influence as much as possible to discountenance it. We do not mean to say that it is an utterly bad practice, or that it is not occasionally of considerable help. With the present dearth of well-educated veterinarians, it is perhaps just now a necessity. We do claim, however, that this method is inherently bad. It leads to the habit of forming vague or careless diagnoses. It makes therapeutics a routine, and a hap-hazard affair generally. The advice that is thus given does no good at all, or does harm quite as often as it benefits the patients. It sometimes is simply a glorification of the editor's particular Spavin-cure, or his Inestimable General Horse-drink or Inimitable Silver Blistering Fluid. It should be remembered also that the correspondents who write the symptoms are persons not trained to observe with any care. Their histories of symptoms must necessarily be incomplete or misleading. It is very well known that in human medicine no diagnosis and no medical advice is ever tendered through the medium of medical journals.

Any thoughtful person who looks over the columns of these veterinary departments will soon be convinced of the weakness of the practice. "My pig," says one, "had fits, or something like them; it got better; then it had another, and died. What was the matter?" No wonder the veterinary editor, having torn his shirt in three places in the mental struggle for a diagnosis, writes, "that the pig had colic, or cerebro-spinal meningitis, or intussusception of the bowels, or inflammation all through the brain." And no wonder that in any case he would give it a Condition Powder, follow that up with a general cow-drink, and put a golden blister at the root of the tail.

Now, perhaps in this particular instance the diagnosis and prescription were correct, and the pig got well, if it had not previously died. But the chances are against it, as they always must be when symptoms originally obscure are imperfectly recorded by those who are utterly ignorant of disease.

We repeat, then, that we hope the veterinary profession will look with disfavor against this custom of newspaper practice. It may be necessary for a time, but let its existence be under a protest, and let it be abolished or reduced to a minimum, as soon as possible.

We ought to say in this connection, however, that most of the veterinary departments which we have examined seem to be conducted with very good judgment and evidence of veterinary skill. The best is made of a bad method.

